# Evolutionary modeling suggests that addictions may be driven by competition-induced microbiome dysbiosis

**DOI:** 10.1038/s42003-023-05099-0

**Published:** 2023-07-26

**Authors:** Ohad Lewin-Epstein, Yanabah Jaques, Marcus W. Feldman, Daniela Kaufer, Lilach Hadany

**Affiliations:** 1 School of Plant Sciences and Food Security, Tel Aviv University, Tel Aviv, 6997801 Israel; 2 Center for Computational and Integrative Biology, Massachusetts General Hospital and Harvard Medical School, Boston, MA 02114 USA; 3 Broad Institute of MIT and Harvard, Cambridge, MA 02142 USA; 4 Helen Wills Neuroscience Institute, University of California, Berkeley, CA 94720 USA; 5 Department of Biology, Stanford University, Stanford, CA 94305 USA; 6 Department of Integrative Biology, University of California, Berkeley, CA 94720 USA; 7 Sagol school of neuroscience, Tel Aviv University, Tel Aviv, 6997801 Israel

**Keywords:** Evolutionary theory, Microbial ecology, Computational models

## Abstract

Recent studies revealed mechanisms by which the microbiome affects its host’s brain, behavior and wellbeing, and that dysbiosis – persistent microbiome-imbalance – is associated with the onset and progress of various chronic diseases, including addictive behaviors. Yet, understanding of the ecological and evolutionary processes that shape the host-microbiome ecosystem and affect the host state, is still limited. Here we propose that competition dynamics within the microbiome, associated with host-microbiome mutual regulation, may promote dysbiosis and aggravate addictive behaviors. We construct a mathematical framework, modeling the dynamics of the host-microbiome ecosystem in response to alterations. We find that when this ecosystem is exposed to substantial perturbations, the microbiome may shift towards a composition that reinforces the new host state. Such a positive feedback loop augments post-perturbation imbalances, hindering attempts to return to the initial equilibrium, promoting relapse episodes and prolonging addictions. We show that the initial microbiome composition is a key factor: a diverse microbiome enhances the ecosystem’s resilience, whereas lower microbiome diversity is more prone to lead to dysbiosis, exacerbating addictions. This framework provides evolutionary and ecological perspectives on host-microbiome interactions and their implications for host behavior and health, while offering verifiable predictions with potential relevance to clinical treatments.

## Abstract (web summary)

A mathematical model of host-microbe dynamics suggests that competition between microbes may promote dysbiosis and increase addictive behaviors, while hosts with diverse microbiomes are more resilient.

## Introduction

Addiction is a brain disease where a victim experiences an uncontrollable motivation to engage in a rewarding behavior despite the behavior’s harmful consequences^1^. Addiction encompasses a range of substance abuse disorders including various drugs, alcohol and cigarettes, as well as excessive food consumption. Such addictive behaviors are among the primary causes of preventable mortality. They are responsible for the deaths of millions of people worldwide each year, while the cost to society is estimated at hundreds of billions dollars. Recent years have witnessed a sharp rise in drug addictions, opioids in particular, with hundreds of thousands of casualties annually from drug overdose worldwide, and 100,000 in the US alone^2–8^.

Recent studies have found various associations between host addictive behaviors, and the composition and functioning of the microbiome, the collection of microorganisms residing in the host^9–13^. These studies accord with a vast body of microbiome research that has revealed pathways by which the microbiome can affect its host’s health and behavior, and others through which the host shapes the microbiome community. Despite these findings, understanding of the ecological and evolutionary processes that shape the host-microbiome ecosystem, and the extent of the microbiome involvement in host chronic diseases and addictive behaviors is still limited.

We hypothesize that microbial strains that are part of the host microbiome may have evolved to affect the host in a way that improves the status of these strains within the microbiome community. This may lead to a community of microbes that affect the host in different directions. Host addictive behavior alters the environmental conditions of its resident microbes. Even if the addiction is largely deleterious to the microbes (e.g., via toxins), for some microbes it may be less deleterious than to others. This generates a shift in the microbial selection regime, and perturbs the microbiome composition^14–18^. Strains that proliferate in the new conditions might benefit from the host continuing its new behavior. Thus, the microbiome may play a role in enhancing and maintaining addictive behaviors.

Addiction is characterized by both negative and positive emotional states attributable, at different stages, to alterations in activity of neurotransmitters: a binge/intoxication stage in which the mesolimbic dopamine system, a key part in the reward circuitry, produces reinforcing actions; a withdrawal stage that is associated with alterations in neurotransmission in the amygdala that generate emotional stress; a preoccupation/anticipation stage in which dysregulation of prefrontal cortex and insula projections interrupts control over incentive salience and therefore goal-directed behavior.

The microbiome has been shown to affect the host brain in multiple ways, for example by modulation of neurotransmitters and interaction with the central nervous system via the gut-brain axis^19–22^. Through this modulation, the microbiome can influence neural activities that are involved in brain reward and withdrawal circuitry by generating negative and positive feedback loops, thus promoting addictive behaviors. For example, neurotransmitters crucial to the functioning of these circuits, such as dopamine, GABA, and serotonin were found to be produced or regulated by the gut microbiome^23–27^. Several studies have shown that gut microbes can synthesize phenylalanine and metabolize L-dopa, both dopamine precursors, and hence they can regulate dopamine levels^28,29^. These processes facilitate microbial pathways that can affect reward circuitry in the brain, for example via dopamine 1 receptors (D1R), which mediate reinforcement and reward, and via dopamine 2 receptors (D2R), which are associated with aversion and avoidance^30,31^. Numerous other mechanisms underlying the microbiome’s impact on host brain and behavior were found, including microbiome-derived short-chain fatty acids^32–34^ and tryptophan metabolism, particularly its role in serotonin synthesis^35–38^. Altogether, this evidence suggests that the microbiome has the potential to affect host states and behaviors through induction of positive and negative reinforcement.

On the other hand, consumption of various addictive substances, including consumption of opioids^39,40^, alcohol^14,15^, smoking^16,41,42^ as well as certain diets^43,44^, has been linked to alterations in microbiome composition. Every such microbiome alteration involves a decrease in the frequency of some microbes, and an increase in the frequency of others. From the perspective of the latter, the host behavior causing the alteration is a beneficial one. We suggest that such host-microbiome interactions, including the host regulating the microbiome composition, and the microbiome modulating the host health and behavior, can form feedback loops that result in major alterations to the host-microbiome ecosystem, inducing and/or aggravating addictive behaviors. Evidence for intricate host-microbiome interactions during addictions, and for their potential role in addiction aggravation, has been found in cases of alcohol consumption and opioid use.

Alcohol consumption has been shown to be associated with major alterations in the gut microbiome in many cases, in both humans and rodents. These alterations include decreased levels of anti-inflammatory bacteria such as *Faecalibacterium prausnitzii* and *Bifidobacterium*, increase of pro-inflammatory bacteria like *Proteobacteria* species, overall reduction in the microbial diversity, increased intestinal permeability, and the release of such inflammatory factors as bacterial peptidoglycans and lipopolysaccharide^14,24,45,46^. Furthermore, it has been shown that such alcohol-induced microbiome alterations significantly correlate with increased striatal D1R expression and reduced striatal D2R expression^24^. It has also been shown that alcoholic patients that did not undergo substantial microbiome disturbances, showed less severe levels of depression, anxiety and craving, and overall experienced milder withdrawal, compared to patients with concurrent microbial changes^47–49^. It is important to note that major alcohol-induced microbiome disturbances were exhibited in many but not all patients without a clear explanation for these occurrences. This may suggest that different microbiome compositions, or even different host-microbiome eco-systems may lead to different addiction and relapse patterns and severity levels.

Opioid addiction is another scenario where the microbiome has been demonstrated to be involved in aggravating addictions. Studies have shown that the microbiome mediates morphine tolerance in mice, promoting addiction. These studies showed that morphine treatment induces microbiome dysbiosis, with selective depletion in *Bifidobacteria* and *Lactobacillaeae*, expansion of Gram-positive pathogenic and reduction in bile-deconjugating bacterial strains^11,50^. Furthermore, it was shown that such morphine-induced microbiome dysbiosis leads to impaired gut epithelia, promoting systemic bacterial translocation and inflammation. The inflammation triggers the induction of proinflammatory cytokines which drive morphine tolerance. These cytokines also aggravate the dysbiosis, hamper the gut integrity, and boost bacterial translocation, thus exacerbating inflammation and reinforcing morphine tolerance^11^. Another study showed that depletion of the gut microbiota resulted in a marked change in behavioral responses to cocaine, causing enhanced sensitivity to its rewarding and sensitizing properties^51^.

Research on microbiome and addictive behaviors is on the rise, yet ecological and evolutionary perspectives of the host-microbiome ecosystem, and their role in addictions, are still missing. Studies have shown that interactions within microbial communities play a prominent role in their functioning and their interactions with the environment, with implications for host health^52–56^. While changes in the microbiome community have been observed in various addiction studies^11,14,15,39,41^, the impact of within-microbiome interactions on host addiction is largely unknown. Previous models studied interactions between microbial strains within the microbiome^53,57,58^ and also host-microbe interactions^59–62^.

Here we combine these two approaches into a framework that models the eco-system of a host, whose wellbeing and behavior shape the microbiome composition, while the microbiome mediates alterations of the host state and behavior. Using this model we analyze several aspects of addictive behavior, focusing on the potential effects of the microbiome on addiction initiation and withdrawal. We show that within-microbiome competition may drive the evolution of microbial feedback on host behavior in a way that improves the microbe’s competitive state but may also exacerbate addictive behaviors. Our model also suggests that microbiome richness and functional diversity are key factors affecting the likelihood of reaching dysbiosis. We find that reduced microbiome richness and diversity lead to a less resilient host-microbiome ecosystem, which is more likely to destabilize and reach dysbiosis, hence aggravating and prolonging the process of behavioral withdrawal and increasing the frequency of relapse episodes.

## Results

We develop a three-component framework for modeling host-microbiome interactions, and their impact on host behavior. First, we model the host behavior as a continuous trait in space. Second, the host contains $N$ microbial strains, each characterized by features that determine its fitness as a function of the host behavior. As the host changes its behavior, the fitness of the different microbial strains also change. Third, we consider the microbiome’s impact on host behavior. We model microbial ability to secrete compounds that affect functions of the host reward and withdrawal circuitry, generating either positive or negative feedbacks (e.g., reward or aversion) that mediate host behavior (see Methods for model description).

### Evolution of microbial feedback to host behavior

We first examine the evolution of a microbial strain that can affect its host’s behavior, by inducing positive or negative feedback, according to the strain’s fitness. If the population of that strain is growing, it secretes compounds that are translated as positive feedback in the host, and if the population is declining, it secretes compounds that are translated as negative feedback in the host. For this purpose, we model the growth dynamics of two microbial strains that reside in a host and compete over resources: one strain affects its host’s behavior, and the second does not. We then extend the model to account for resource-competition dynamics among numerous microbial strains that can affect host behavior and investigate host-microbiome interactions, focusing the analysis on host addictive behavior (see Methods).

**Fig. 1:**
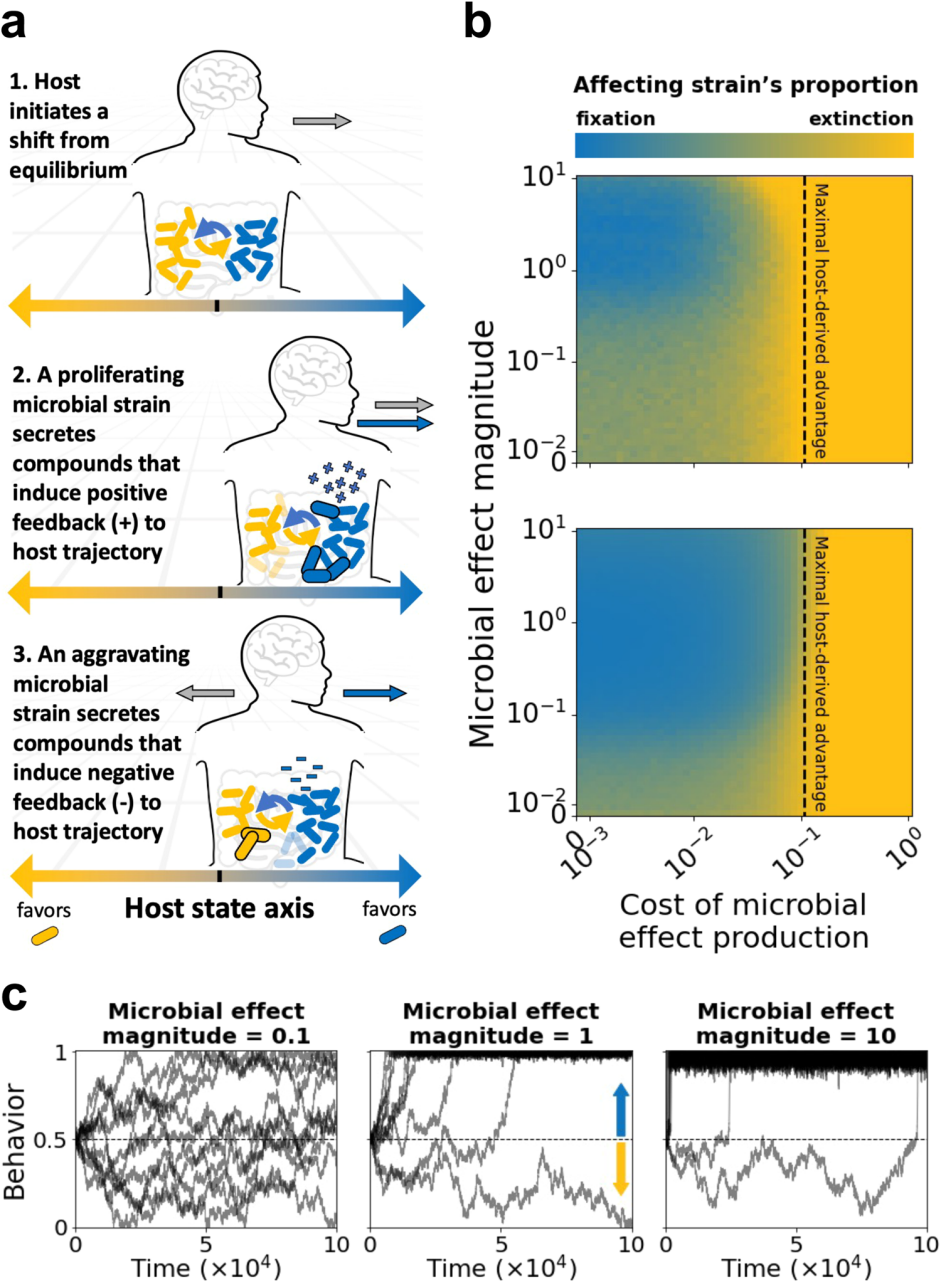
A microbe’s effect on its host’s state may be beneficial when it provides an advantage over a competitor that outweighs the cost of producing that effect. **a** Model Illustration. We model competition between two microbial strains for host resources, which are derived from the host behavior. Host behavior is modeled as a random walk along the [0,1] interval, starting at 0.5. The microbial strains are represented by coordinates on that same segment, while the distance between their coordinates and the behavior coordinate represents the fitness of each strain under that host behavior (see Methods). One of the two microbial strains (blue) has the potential to affect its host’s behavior: it secretes positive feedback when proliferating, inducing the host to continue its behavioral trend, and secretes negative feedback when declining, inducing the host to reverse its behavioral trend. These secretions are proportional to the strain’s abundance (see Methods). **b** Heatmaps presenting the proportion of the strain that affects its host, after 100,000 time points, as a function of the effect’s magnitude and the cost of producing the effect, for two different intra-strain competition regimes (upper panel: $s=0.01$; bottom panel: $s=0.1$). The vertical dashed lines represent the maximal advantage that the affecting strain can gain from the host behavior, relative to its competitor (0.1 in these simulations); thus it is not expected to succeed when the cost is greater (see Methods). Each pixel represents the average result of 500 simulations. **c** Time series examples showing the host behavior as function of time over 100,000 time points; $s=0.1$ and the cost of effect production is 0.09. In panels (**b**) and (**c**) the coordinates of the affecting strain and its non-affecting competitor were set to 0 and 1 respectively. The line at 0.5 is the starting position of the behavior. See Methods for a detailed description of the model.

We find that a microbe that provides feedback to host behavior may be selectively favored over a wide parameter range, including when it suffers a cost for producing this feedback. Such microbial effects would be favored when the additional resources the microbe gains from the altered host behavior, either directly or through the competition with other strains, compensate for the costs required to produce the effects. Moreover, once the affecting strain manages to draw its host towards a behavior that is more beneficial to that strain, its proportion in the microbiome rises and so does its ability to continue affecting the host, further inducing the host to continue the new behavior (Fig. 1).

### Host-microbiome interactions may exacerbate addictive behaviors

**Fig. 2:**
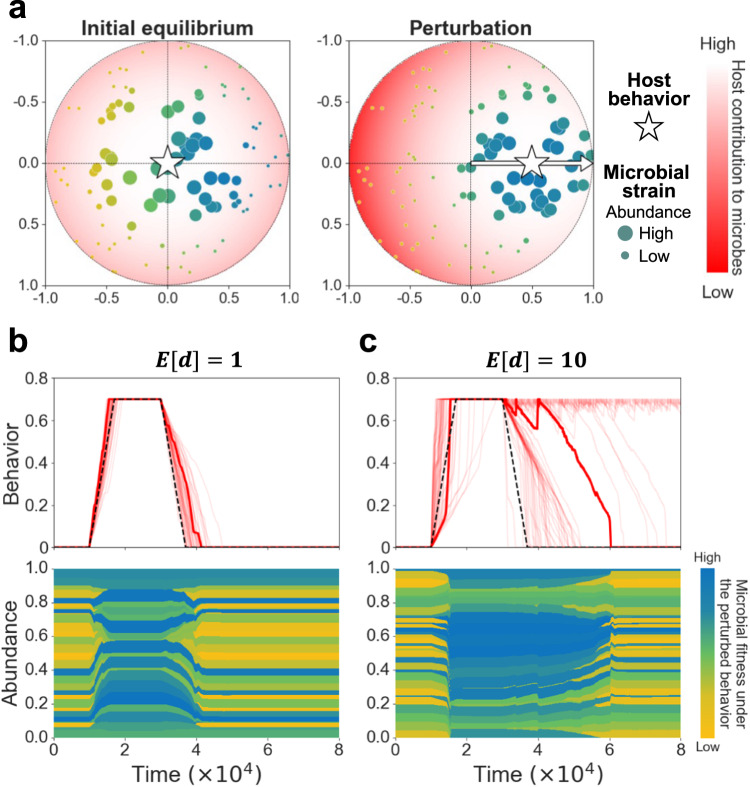
Microbiome effect on host behavior may considerably decelerate the withdrawal stage. **a** Model illustration. The host behavior is represented by coordinates in the 2D unit sphere (star). Microbial strains (colored dots) are characterized by features, represented as coordinates in that sphere. The host contribution to the growth of each strain is a function of the distance between the host behavior and the strain’s features (see Methods). Thus, the microbial coordinates represent the access to host-derived resources, as a function of the host behavior. The illustration demonstrates how a perturbation in the host behavior (movement of the star) produces a change in the contribution of the host to each microbial strain, which affects the microbiome composition. The color and size of each dot represents the strain’s feature-distance from the perturbed host behavior (bluer is closer) and the strain’s proportion within the microbiome, respectively. **b**, **c** Simulation examples. Upper panels show a collection of simulation results: the change in behavior over time without (dashed black) and with microbiome effects (red; 50 runs in each panel), for different mean microbiome effect magnitudes $\left(E\left[d\right]=1,\,10\right)$. The bold red curve represents a randomly selected simulation run, for which the microbiome composition over time is plotted in the bottom panels. Each stripe (yellow-blue scale) represents a microbial strain, while the width of the stripe represents the temporal proportion of the strain within the microbiome. As in panel (**a**), the color of each stripe represents the strain’s feature-distance from the perturbed host’s behavior, which is the basis for the fitness evaluation. The number of strains $\left(N\right)$ is set to $50$.

We employ this framework to study the potential effect of the microbiome’s behavioral feedback on host addictions. Expanding our initial model, we consider a microbiome comprising $N$ microbial strains, with features modeled as 2D-coordinates in a unit-sphere. We chose two dimensions for convenience of presentation, and later investigate the impact of varying this. We assume that the microbes compete with each other for resources from the host, and that each microbial strain has the potential to affect its host’s behavior (Fig. 2a). In each simulation we randomly assign to each strain its coordinates (uniformly across the unit-sphere) and its effect on the host (denoted by ${d}_{i}$ for strain $i$; sampled from a distribution with mean $E\left[d\right]$; see Methods).

We model a simple host behavioral pattern, consisting of three stages. First, the host begins at a certain equilibrium. Second, the host gradually changes its behavior until it reaches some peak behavior level ($0 < R < 1$), where it stays for a period. Third, the host gradually moves back towards its initial behavior. This behavioral pattern serves as a baseline to which we compare the impact of the microbiome. Its simplicity enables a focused analysis on how host-microbiome interactions can alter host behavior, giving rise to addictive- and withdrawal-like behaviors. We demonstrate here how host-microbiome co-regulation can impact the host reward circuitry, and can serve as another factor exacerbating addictions, in addition to other well-studied factors^63–65^. We thus term the period of the initial change in behavior *Addiction*, and the period of the reverse behavior as *Withdrawal*. We examine a behavioral pattern that follows a path from the center of the space (the initial equilibrium) towards the boundary. Since the microbial features are randomly chosen within the space, without losing generality we set the domain of the host behavior to be the [0,1] segment along the *x*-axis. This enables the host behavior-coordinate to measure the severity of the behavioral-alteration, namely the addiction, or the degree of consumption of an addictive substance, and its effect on the microbiome.

We find that intra-microbiome competition, combined with microbial effects on host behavior, can lead to either acceleration or deceleration of the addiction process, while exacerbating the withdrawal phase under a wide range of conditions. We assume that before the addiction begins, the host maintains a rich and diverse microbiome. Thus, when the host initiates a change in its behavior, equal numbers of microbial strains are expected to favor or disfavor the behavioral change. However, as the change in the host behavior continues, microbial strains that are favored by the altered conditions increase in proportion, and their feedback effect becomes stronger, inducing the host to continue in its trajectory. Accordingly, we find that the microbiome’s effect on host behavior can lead both to acceleration and deceleration of the addiction, depending on the distribution of microbial features and the magnitudes of their effects. In contrast, we find that the microbiome behavioral effect decelerates the withdrawal stage in most cases. This is because during the addiction the microbiome shifts towards a less diverse community, made up of the strains that proliferate under the new host behavior. Thus, when the withdrawal stage is initiated by the host, a considerable proportion of the microbiome resists the behavioral change, decelerating the withdrawal across a very wide range of conditions (Fig. 2b, c). As in many models of complex systems, this kind of host-microbiome interaction can generate a positive feedback loop that drives the system out of balance, and in severe cases it crosses a tipping point^66,67^.

### The effect of the microbiome richness on addictive behaviors

**Fig. 3:**
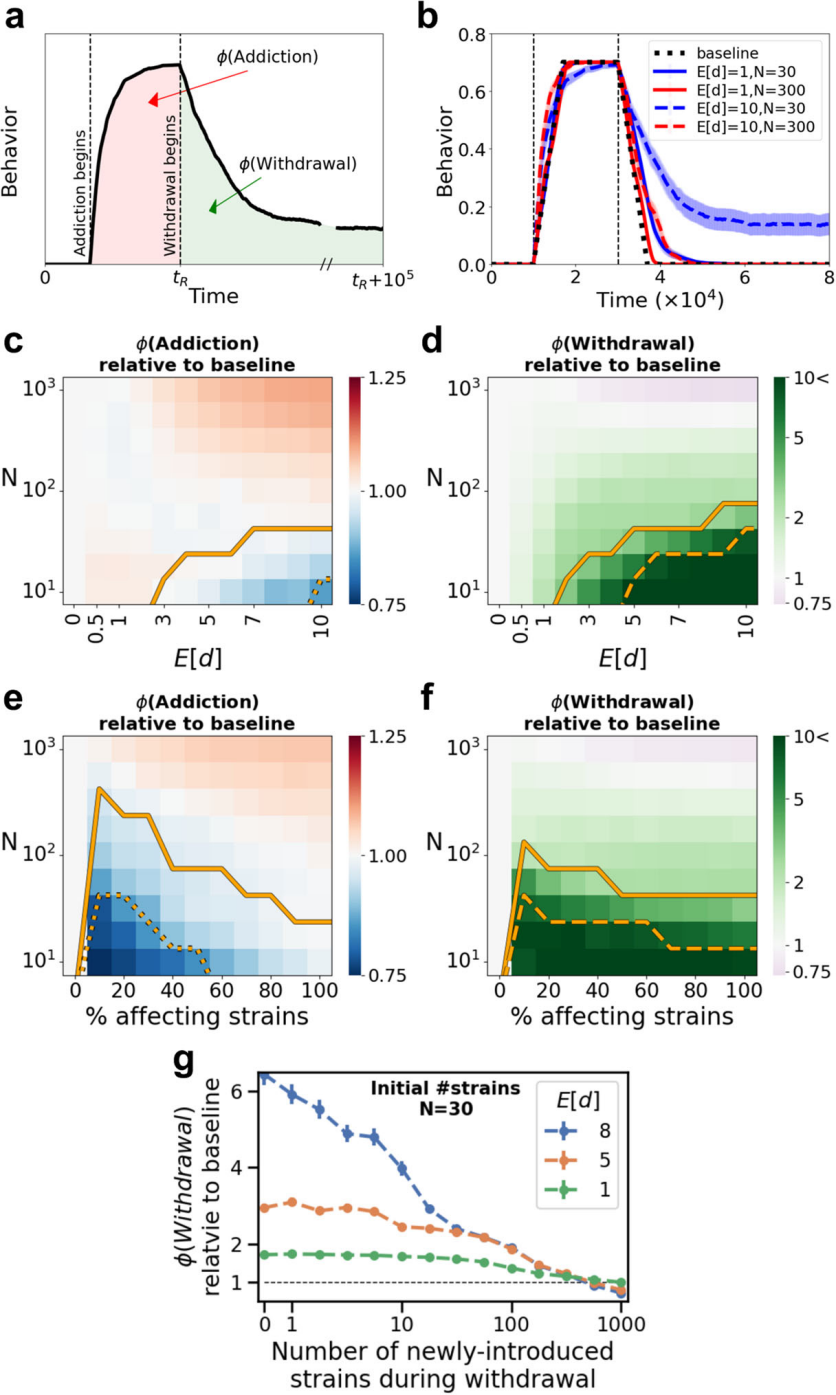
Low richness of the host microbiome may affect the addiction process and lead to substantial aggravation of the withdrawal stage. **a** Illustration of a host-behavior time series. The colored markings present the integral of the behavior over time, for the addiction process ($\phi$(Addiction); light red) and withdrawal process ($\phi$(Withdrawal); light green). For the addiction process we consider the time from the addiction initiation until the withdrawal initiation, while for the withdrawal we consider the time from the withdrawal initiation, until the end of the simulation, 100,000 time points after withdrawal stage initiation. **b** Average host behavior over time is plotted for the baseline case of no microbiome effect (dashed black) and for cases that include the microbiome effect (red-blue curves), considering two mean microbial effect magnitudes $\left(E\left[d\right]\right)$ and two numbers of available strains $\left(N\right)$. Each curve presents the average of 50 simulation runs. **c**, **d** The color of each pixel in the heatmaps represent the fold increase or decrease in $\phi$(Addiction) (**c**) and $\phi$(Withdrawal) (**d**) relative to the baseline case of no microbiome effect, as functions of $N$ and $E\left[d\right]$. Each pixel in the heatmap presents the average of 1000 simulations (see also Methods and Supplementary Fig. 6). **e**, **f** The color of each pixel in the heatmaps represent the fold increase or decrease in $\phi$(Addiction) (**e**) and $\phi$(Withdrawal) (**f**) relative to the baseline case of no microbiome effect, as a function of $N$ and of the percentage of strains that can affect the host behavior. Each pixel in the heatmaps presents the average of 1000 simulations. To keep the mean of the overall manipulation strength of the microbiome constant and vary only the proportion of strains that affect the host behavior, we set the mean magnitude of the microbes’ effects $E\left[d\right]=\frac{5}{{proportion\; of\; affecting\; strains}}$. Below the solid lines in (**c**) and (**e**), in more than 1% of the simulations the behavior does not reach the maximal addiction severity $\left(R\right)$, and below the dashed line, in more than 20% it does not. Below the solid line in (**d**) and (**f**) the behavior does not return to the initial state at the end of the simulation in more than 1% of the runs; and below the dashed line, the behavior does not return in more than 20% of the runs. **g** The $\phi$(Withdrawal) relative to the baseline case of no microbiome effect is plotted as function of the number of new strains that are introduced to the system during the withdrawal stage, for several mean microbial effect magnitudes $\left(E\left[d\right]\right)$. For the analysis of this panel the initial microbiome community, before the introduction of new strains during the withdrawal, was set to contain 30 different strains. Each dot represents the average of 1000 simulations. Error bars represent the standard error of the mean. $R=0.7$ is used throughout the analysis for this figure.

In order to study the effects of microbiome richness on the addiction and withdrawal processes, we examine a wide range of numbers of microbial strains in the system, from 10 to 1000. The characteristics of each microbial strain are represented by two parameters, chosen at random: first, its ability to affect the host behavior; second, the strain’s change in proliferation resulting from the host behavior. Hence, each strain in our model can represent an arbitrary taxonomic classification, from different phyla (~10 in the human microbiome^68^) to different species (up to 1000 in the human microbiome^69,70^), or more generally, classification into units that share specific attributes, regardless of the phylogeny. To track not only the overall duration of these processes but also the quantitative changes in behavior, we denote the integral of the behavior level over time for each phase by $\phi \left({Addiction}\right)$ and $\phi \left({Withdrawal}\right)$, (Fig. 3a), and monitor them. We find that microbial richness has an effect on the withdrawal stage: withdrawal decelerates with decreasing microbiome richness, and with increasing magnitude of the microbiome effect. With respect to the addiction process, the effect of microbiome richness $\left(N\right)$ depends also on the mean magnitude of the microbiome effect ($E\left[d\right]$; see Fig. 3b–d). In cases of very high microbial richness and strong microbial effects, the microbiome may actually hasten both addiction and withdrawal (upper rows of Fig. 3c, d).

An essential contributor to the addiction-aggravation dynamics is the competition between the microbial strains over host resources. Without competition, the strains will grow at rates that depend on the host state, but will eventually reach the same carrying capacity abundance, regardless of the host state. Hence, host-microbiome feedback would not form. The competition is represented in our model by keeping the microbial population at carrying capacity, assuming limited host-resources (see Methods). Thus, even if a certain strain encounters somewhat stressful conditions, it may still propagate in the population, if these conditions are more stressful for the rest of the community. On top of these resource-competition dynamics, community diversity plays a critical role in shaping the host-microbiome interactions. Diversity is represented by the multiple niches that the bacteria can occupy, in the context of their favored conditions in terms of host status. A less diverse microbiome is more prone to be tilted towards a new equilibrium, from which it is harder to return to the initial state. When the microbiome is more diverse, changes are easier; for any change in host behavior a substantial number of strains benefit, thus reinforcing the host’s behavioral trajectory.

Although the space of host states and microbial features could be high dimensional, we find that the results do not change qualitatively when varying the number of dimensions. As expected, increasing the number of dimensions leads to increased sparsity of the microbial strains in the behavior-fitness space; thus the impact on the addiction and withdrawal is similar to decreasing the number of microbial strains $\left(N\right)$ in the model (Supplementary Fig. 1).

We also investigate the case where only some of the strains affect host behavior by examining the impact of the distribution of microbial effects on host behavior. We first vary the proportion of strains that affect host behavior, while keeping the average of the total microbiome effect constant. Distributing the effects among fewer microbial strains results in deceleration of both addiction and withdrawal (Fig. 3e, f). Fixing the mean magnitude of microbial effects, then decreasing the proportion of affecting strains, results in a decrease in the overall microbiome effect and an accelerated withdrawal process. Nevertheless, even when only a minority of the strains affect host behavior, the impact on addiction and withdrawal can be substantial (Supplementary Fig. 2). We also examine the effect of applying various levels of costs imposed by the microbial effects, and setting a constant host effect on the growth of some of the microbial strains (rather than allowing behavioral dependence); our model was robust to these changes (Supplementary Figs. 3–5).

Moreover, an intervention that includes increasing the microbiome richness and diversity during the withdrawal stage can mitigate the withdrawal and shorten its duration. We examine this by initiating the simulations with a low-richness microbiome composition $\left(N=30\right)$, and when the withdrawal stage begins, new microbial strains with randomly assigned features, are introduced to the system (Fig. 3g). This type of intervention gradually increases the inter-strain competition within the microbiome and decreases the impact of the addiction-adapted strains, thus facilitating the shift of the host-microbiome ecosystem towards its original equilibrium.

### The impact of addiction severity

**Fig. 4:**
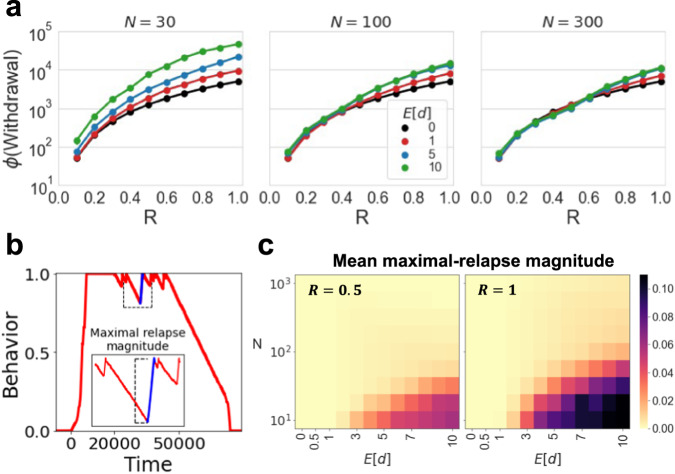
Exacerbating effect of microbiome on host withdrawal increases with addiction severity. **a** The integral of the behavior over time during the withdrawal stage ($\phi$(Withdrawal)) is plotted as a function of the maximal addiction severity $\left(R\right)$, for several numbers of available strains $\left(N\right)$ and the mean magnitudes of the effects $\left(E\left[d\right]\right)$. Each dot represents the average of 1000 simulations. **b** Relapse schematic example: we define a relapse as an increase in the addictive behavior that occurs after the withdrawal phase has begun. In each simulation we define the maximal-relapse magnitude as the maximum among the differences between all coordinates of the behavior in the withdrawal phase, and the coordinates of behavior that follow. **c** The color of each pixel in the heatmaps represent the mean maximal-relapse magnitude as a function of $N$ and $E\left[d\right]$, for different maximal addiction intensities. Each pixel in the heatmaps presents the average of 1000 simulations.

Finally, we examine the effect of the microbiome’s interaction with the maximal severity of addiction ($R$), which represents the maximal impact of host behavior on the microbiome: higher values mean that the host can reach a state in which microbiome compositions are more distant from the initial equilibrium. We find that as the addiction becomes more severe (higher $R$), the host behavior generates an ecological regime that leads the microbiome towards a narrower niche with lower diversity. After establishment, the new microbiome composition may strongly reject any attempt to make a change, thus slowing down the withdrawal process. This dynamic increases with the magnitude of the effect of the microbiome on host behavior, and decreases with microbiome richness. When the microbiome is richer and/or its effect on the host is relatively weak, only a substantial alteration in the microbiome composition results in an aggravation of the addiction (Fig. 4a). We also see an effect of the maximal addiction severity $\left(R\right)$ on occurrences of microbiome-induced relapses. We define a relapse as an aggravation in the addictive behavior (increase in the behavior coordinate) that occurs during the withdrawal phase (Fig. 4b). We see that stronger microbial effects, lower microbiome richness, and higher addiction severity all result in stronger and more frequent relapses (Fig. 4c).

## Discussion

We hypothesize that within-microbiome competition may lead to evolution of microbial effects on host behavior, and that these effects could play a role in host addictive behavior. Our results demonstrate that microbiome feedbacks to host behavior can aggravate addictive behaviors, making withdrawal attempts more difficult and leading to higher risk of relapses. Microbiome richness is a key parameter in the process, with low richness resulting in prolonged addictions.

Our framework includes both within-microbiome competition over resources and host-microbiome interactions, and can be extended in several ways. Other microbial interactions can be incorporated including direct competition (e.g., toxins), cooperation (e.g., cross-feeding) and exploitation. In addition, the interactions between host and microbiome can be modeled by more complex functions, e.g., threshold functions or non-monotonic functions, including rugged landscapes^71,72^. Different manifestations of host-exerted selection can be modeled. While in our model the host state affects each of the strains’ growth rate, it could also affect each of the strains’ carrying capacity, leading to a similar form of host-microbiome feedback loop. Furthermore, several different forms of microbial effects on the host behavior can be investigated, including specific reactions to certain host behaviors^11^ (e.g., microbial reaction to exposure to opiates leading to secretion of compounds that generate host tolerance and hence induce addiction). Our model assumes a more general interaction, grounded in empirical evidence, where the microbes can affect the host by providing feedback—either positive or negative—on host behavior, through modulation of the reward circuitry. In addition, we consider a simple model of host addictive behavior, and focus on the effect of the microbiome on the process. Potential extensions may also include integrating host-brain mechanisms of addiction (reward sensitization, associative learning, conditioned reinforcers, impulse control, etc.), where the microbiome is able to affect these processes, as well as short-term and long-term effects of the addiction from both host and microbiome perspectives.

Much work has been devoted to studying the stability of communities and how stability is affected by community richness and internal interactions such as cooperation and competition^57,73–75^. Although our work addresses these ecological factors, it is quite different from the traditional literature on these topics. First, we focused on host-microbiome interactions, and in particular on the microbe-induced changes in the environment. Second, we do not study the stability of the microbiome community according to the classical response of the community to a perturbation. Rather, we examine the dynamics during and after a gradual and prolonged host-induced alteration. Third, we do not consider direct interactions, negative or positive, between the interacting strains, but only resource competition.

In our model, the microbial effect on host behavior can be considered a form of public goods—microbial secreted products that benefit the strains that proliferate in the new host-mediated conditions. Numerous studies have investigated public goods secretions in microbial communities, demonstrating that such behaviors can be maintained in a population despite their direct cost to the secreting microbe, while also analyzing the conditions for the evolution of such behaviors^76–78^.

Our results accord with empirical evidence. First, it has been demonstrated that the microbiome can alter neuronal activity, modulating factors that regulate the reward circuitry in the brain through reinforcement and aversion^28,29,35–38^. Moreover, these microbiome mechanisms are involved in addictions to alcohol and opioids^24,45,47–49,51^. Second, empirical studies have found that in response to opioid uptake, host-microbiome interactions generate a positive feedback loop that alters the ecosystem and exacerbates the addiction^11^. Finally, studies have also showed that a less rich and diverse microbiome is associated with aggravated addiction^14,51^. Despite this large literature, our model is, as far as we know, the first attempt to suggest evolutionary and ecological perspectives for microbiome-induced addictive behaviors.

Targeting the microbiome may reveal additional avenues for addiction treatments. Our model predicts that increased microbiome richness and functional diversity could contribute to addiction mitigation and prevention, and suggests that hosts with very low microbiome richness may be at greater risk for addiction and for relapse during the withdrawal period. In this context, stress and anxiety are factors that are associated with both low microbiome diversity and host addictive behaviors^79–82^, suggesting that the interplay between host physical and mental state, microbiome composition, and addictions would be interesting to investigate. Future experiments will be required to decipher the mechanisms underlying microbiome involvement in host addictive behavior and brain function. Investigating the microbes that may benefit from an addiction may reveal potential candidates for interventions.

The framework presented here can be generalized and used to investigate other host-microbiome interactions with various implications on host behavior and wellbeing. For example, our framework could be relevant for studying host-microbiome interactions with respect to the host immune system, where the immune system of the host shapes the microbiome composition, and the microbiome affects the development and maintenance of the immune system^83,84^. Moreover, this framework can be used for modeling complex ecological systems, incorporating numerous species that compete with each other, as well as environmental factors that affect the competition dynamics between these organisms, but are also altered by them^85^. In this context, our model can be considered as a form of niche construction^86^, where a community of species acts to construct a favorable niche in a certain environment; in our case, the environment is itself an organism.

Better understanding of host-microbiome interactions with respect to addictive behaviors may uncover additional mechanisms behind addictions and generate strategies for treatment. Our results call for empirical studies—characterizing within-microbiome interaction networks, uncovering mechanisms of microbial effects, and testing the association between microbiome richness and functional diversity on the one hand and addictive behavior on the other.

## Methods

We model a host interacting with its microbiome, where the host behavior affects the microbiome composition and the microbiome affects the host behavior. We consider two models using the same framework. The first depicts the potential advantage of microbes’ ability to affect their host’s behavior, by modeling the growth dynamics of two microbial strains residing in a host: one strain affects its host behavior, and a second strain does not. The second addresses the potential role of microbial effects on addiction, accounting for a microbiome community that comprises $N$ microbial strains, where some or all of the strains can affect the host’s behavior.

### Host baseline behavior

The host baseline behavior is represented as coordinates (denoted by $\vec{b}$) in a two-dimensional unit-sphere, termed the microbiome-behavior-space. The host’s baseline behavior changes over time according to a pre-defined pattern. This baseline serves as a null model that represents the host behavior free of the microbiome’s effect. Later, the microbiome effect is added to this baseline.

For the two-strain competition, the host baseline behavior is modeled as a random walk along the segment $\left[{{{{\mathrm{0,1}}}}}\right]$ on the *X*-axis of the microbiome-behavior-space; this can represent, for example, the consumption of a chemical resource. Each simulation starts in the middle of that range, at 0.5. The step at each time point $t$, denoted by ${\sigma }_{{RW}}\left(t\right)$ is randomly drawn from a double-exponential (Laplace)^87,88^ distribution with mean $0$ and scale $\sigma .$

In the simulations that yielded the results for Fig. 1, $\sigma \,{{{{{\rm{was}}}}}}\; {{{{{\rm{set}}}}}}\; {{{{{\rm{at}}}}}}\,{10}^{-3}$.

For the addiction model, the analysis focused on host baseline behavior that follows a simple addiction scenario, including three behavioral phases (Fig. 2b):

**Initial equilibrium**. $\vec{b}=\vec{0}$. This phase ends when the host’s microbiome composition stabilizes.

**Addiction**. Alteration in the baseline behavior. The change in the host baseline behavior is simulated as a series of positive movements along the *X*-axis. At each time point $t$, a step size, denoted by ${\sigma }_{A}\left(t\right)$, is randomly drawn from an exponential distribution with mean $\sigma$. The change in the host behavior is bounded by distance $R$ from the origin, representing the maximal addiction severity. Thus, the host-behavior coordinates shift gradually from $\left({{{{\mathrm{0,0}}}}}\right)$ up to $\left(R,0\right)$, and stay at $(R,0)$ until the end of this phase, $\tau$ time steps after its initiation.

**Withdrawal**. The host reverses its behavioral pattern, by moves of $\left(-{\sigma }_{A}\left(t\right)\right)$ on the X-axis, with ${\sigma }_{A}\left(t\right)$ randomly drawn from exponential distribution with mean $\sigma$. The change in behavior is bounded below by 0. This phase ends when the microbiome composition and host behavior stabilize, or after 100,000 time steps from the initiation of the phase.

Unless stated otherwise, *σ* was set 10^−4^ and $\tau$ was ${{{{\mathrm{20,000}}}}}$.

### Microbiome composition

A population of *N* microbial strains inhabits a host. Each strain has its unique features, modeled as coordinates in the microbiome-behavior-space. We denote the vector of these coordinates for each strain $i$ by $\vec{{m}_{i}}$, and the growth dynamics of the different microbial strains are based on the common system of ordinary differential equations that describes the change in the frequency of strain $i$ over time^57,73^:

1 $$\frac{d{x}_{i}}{{dt}}={x}_{i}\left(t\right)\cdot \left({r}_{i}-{s}_{i}\cdot {x}_{i}\left(t\right)\right)$$

The forward Euler method was used to simulate the dynamics of this system of equations, with arbitrary time steps of size 1, which defines the system in discrete time:

2 $${x}_{i}\left(t+1\right)={x}_{i}\left(t\right)+{x}_{i}\left(t\right)\cdot \left({r}_{i}\left(t\right)-{s}_{i}\cdot {x}_{i}\left(t\right)-{c}_{i}\right)$$

where ${x}_{i}\left(t\right)$ denotes the proportion of strain $i$ in the microbiome at time $t$; ${r}_{i}\left(t\right)$ denotes the growth rate of strain $i$ at time $t$, which is a function of the host behavior (see below); ${s}_{i}$ denotes intra-strain competition; and ${c}_{i}$ denotes the constant cost of feedback production experienced by strain $i$.

In order to incorporate resource competition among the different microbial strains in the microbiome, we normalized the ${x}_{i}\left(t+1\right)$ values by their sum at each iteration. Thus the microbial strains’ relative abundance is tracked, while assuming a constant total microbiome abundance, similar to having a carrying capacity for the entire microbiome community. We also include a constant low inflow of all $N$ microbial strains at rate $\frac{\mu }{N}$ for each strain; thus our results do not rely on extinction and permanent disappearance of strains from the system ($\mu$ was set at ${10}^{-8}$ throughout). At each time step, the strain proportions are calculated using Eq. (2), followed by inflow and a second normalization. In the simulations that yielded the results for Figs. 2–4 and the supplementary figures, $s$ was set at 1. For Fig. 1 we used $s={{{{\mathrm{0.1,0.01}}}}}$. The effect of ${c}_{i}\ne 0$ is shown in Fig. 1 and in Supplementary Figs. 3–4; ${c}_{i}$ was set at 0 in the simulations that yielded the rest of the results. We conducted two additional analyses, one where the per-strain inflow rate is the same regardless of the number of strains, and a second where there is no inflow; the results were quite similar (Supplementary Figs. 7–8).

For the two-strain competition model $\left(N=2\right)$ the features of each strain are manually defined and specified in the results. One strain affects the host behavior, and this strain pays the cost of feedback production, while the other strain does not affect the host behavior and does not pay the cost.

For the addiction model the features of each strain are drawn randomly within the microbiome-behavior-space (from a uniform distribution in the 2D unit sphere), at the beginning of each simulation. We consider a microbiome community that comprises $N$ microbial strains, where some or all of the strains can affect the host behavior.

A generalized Lotka-Volterra model that did not include normalization was also analyzed (also using the forward Euler method with arbitrary time steps of size 1):

3 $$\frac{d{x}_{i}}{{dt}}={x}_{i}\left(t\right)\cdot \left({r}_{i}\left(t\right)+{A}_{i}\cdot x\left(t\right)\right)+\frac{\mu }{N}$$

This system of equations is similar to the procedure mentioned above, with two main modifications. First it does not include normalization steps. Second, it includes matrix $A$, (${A}_{i}$ is row $i$ in the matrix), in which each element $\left({a}_{{ij}}\right)$ represents the impact of strain $i$ on the growth of strain $j$. The diagonal of $A$, is set to -1, modeling intra-strain competition, similar to the main model. We set all other elements of $A$ $\left(i\ne j\right)$ to be $-\delta$, representing a scenario similar to the main model, where the growth of each strain negatively affects the growth of all other strains. This model yields qualitatively similar results to those obtained using Eq. 2 (Supplementary Fig. 6).

### Host-behavior effect on the microbiome composition

We focus on aspects of host behavior that regulate the ecology of the microbiome and thus affect the microbiome composition. The host-derived resources obtained by each strain are determined by a monotonically decreasing function of the Euclidean distance between the host and the microbial strain, in the microbiome-behavior-space. In this context the microbial features relevant for the host-microbe interaction are the focus. We define ${r}_{i}$ at time $t$ as

4 $${r}_{i}(t)=0.1+{{{{{\rm{max }}}}}}\left(0,1-\beta \cdot {\left({||\vec{b}\left(t\right)-{\vec{m}}_{i}||}_{2}\right)}^{\alpha }\right)$$

Unless stated otherwise, we used $\alpha =3,\,\beta =0.1.$ Supplementary Fig. 9 demonstrates the robustness of our results to these parameters. The effect of setting a constant host effect on the growth of some microbial strains was also examined (rather than having the effect be behavior-dependent), and our model was robust to this change (Supplementary Fig. 5).

### Microbiome effect on host behavior

We assume that all or some of the microbial strains can affect their host’s behavior and that the microbes can sense beneficial and deleterious changes in their population, portrayed as temporal increase or decline in their population size. When a strain’s population is increasing, the microbes produce and secrete compounds that are perceived by the host as positive feedback (e.g., reward), and when the population is declining the microbes produce and secrete compounds that are perceived by the host as negative feedback (e.g., aversion). These feedbacks are then integrated by the host, affecting the future behavioral trajectory. We denote by ${B}_{i}^{{\omega }_{m}}\left(t\right)$ the slope of the linear regression on the proportions of strain $i$ in the past ${\omega }_{m}$ time points:

5 $${B}_{i}^{{\omega }_{m}}\left(t\right)=\frac{{\sum }_{j=t-{\omega }_{m}}^{t}\left(j-{\bar{j}}^{{\omega }_{m}}\right)\left({x}_{i}\left(j\right)-{\bar{x}}_{i}^{{\omega }_{m}}\right)}{{\sum}_{j=t-{\omega }_{m}}^{t}{\left(j-{\bar{j}}^{{\omega }_{m}}\right)}^{2}}$$

where ${\bar{j}}^{{\omega }_{m}}$ is the average of the time-point indicators $\left(t-{\omega }_{m},{t}-{\omega }_{m}+1,\ldots ,t\right)$, ${x}_{i}\left(j\right)$ is the proportion of strain $i$ at time point $j$, and ${\bar{x}}_{i}^{{\omega }_{m}}$ is the average of strain’s $i$’s proportions during the time period $t-{\omega }_{m}$ through $t$. We then denote by ${I}_{{m}_{i}}\left(t\right)$ the condition of microbial strain $i$ at time $t$, defined as follows:

6 $${I}_{{m}_{i}}\left(t\right)=\left\{\begin{array}{cc}1 & {B}_{i}^{{\omega }_{m}}\left(t\right) \, > \, {10}^{-6}\\ -1 & {B}_{i}^{{\omega }_{m}}\left(t\right) \, < \,{-10}^{-6}\\ 0 & {else}\end{array}\right.$$

Here, the condition of strain $i$ is positive, negative, or neutral (therefore it secretes positive feedback, negative feedback, or no feedback at all) depending on the slope of the linear trajectory of its proportion in the past ${\omega }_{m}$ time points. Slopes that are very close to zero (between ${-10}^{-6}$ and ${10}^{-6}$) are considered neutral in their effect, representing accuracy limitations in evaluation. These feedbacks affect the host behavioral change later on.

We assume that the host associates its recent behavioral trend with integrated microbial feedbacks. If the recent trajectory is associated with positive feedback, the host will continue with the same trajectory. If the recent trajectory is associated with negative feedback, the host will reverse its behavioral trajectory.

We denote by ${B}_{{host}}^{{\omega }_{h}}\left(t\right)$ the slope of the linear regression on the host behavior in the past ${\omega }_{h}$ time points (considering its moves along the *X*-axis; similarly to the calculation in the previous paragraph, except that the regression is of the host-behavior coordinate along the *X*-axis). We then denote by ${I}_{b}\left(t\right)$ the behavioral trajectory of the host at time $t$, as follows:

7 $$\,{I}_{b}\left(t\right)=\left\{\begin{array}{cc}1 & {B}_{{host}}^{{\omega }_{h}}\left(t\right) \, > \,{10}^{-6}\\ -1 & {B}_{{host}}^{{\omega }_{h}}\left(t\right) \, < \, -\!\!{10}^{-6}\\ 0 & {else}\end{array}\right.$$

Hence, the host trajectory—away from the center, towards the center or neutral—depends on the slope of its linear trajectory over the past ${\omega }_{h}$ time points.

Unless otherwise stated we set ${\omega }_{h}={\omega }_{m}=10.$ Supplementary Fig. 10 demonstrates the robustness of our results to these parameters.

Combining the microbial secretions and the behavior trajectory, the direction of microbe $i$’s effect on the host behavior at time $t$ can be described by the sign of the product ${I}_{{m}_{i}}\left(t\right)\cdot {I}_{b}\left(t\right)$. This term is positive when strain $i$ influences the host to advance on the addiction path (e.g., to increase doses of the consumed substance), in one of two scenarios: strain $i$ provides positive feedback $({I}_{{m}_{i}}\left(t\right)=1)$ to host advancement on the addiction path $\left({I}_{b}\left(t\right)=1\right)$, or strain $i$ provides negative feedback $({I}_{{m}_{i}}\left(t\right)=-1)$ to host withdrawal $\left({I}_{b}\left(t\right)=-1\right)$. The term ${I}_{{m}_{i}}\left(t\right)\cdot {I}_{b}\left(t\right)$ is negative when strain $i$ influences the host to take a step backwards on the addiction path (e.g., to reduce consumption of the substance); and when it is zero, strain $i$ does not affect host behavior.

The total effect-strength of each strain, at time $t,$ is that strain’s proportion within the microbiome, multiplied by$\left(\left|{\sigma }_{A}\left(t\right)\right|\cdot {d}_{i}\right)$, where ${d}_{i}$ denotes the magnitude of the effect of strain $i$ on the host, and $\left|{\sigma }_{A}\left(t\right)\right|$ is the baseline behavioral step size at time $t$. The microbial effect magnitudes $\left({d}_{i},{i}\in \left\{1,\ldots ,N\right\}\right)$ are chosen at random from an exponential distribution with mean $E\left[d\right]$ at the beginning of each simulation, and ${\sigma }_{A}\left(t\right)$ are drawn at each time step, as explained above.

The effect of the entire microbiome at time $t$, denoted by $M\left(t\right)$, is the sum of all strains’ effects:

8 $$M(t)=\mathop{\sum }\limits_{i=1}^{N}(\overbrace{{x}_{i}(t)}^{proportion}\cdot \overbrace{|{\sigma }_{A}(t)\cdot {d}_{i}|}^{effect\,size}\cdot \overbrace{({I}_{{m}_{i}}(t)\cdot {I}_{b}(t))}^{effect\,direction})=|{\sigma }_{A}(t)|\cdot {I}_{b}(t)\cdot \mathop{\sum }\limits_{i=1}^{N}({x}_{i}(t)\cdot {d}_{i}\cdot {I}_{{m}_{i}}(t))$$

The change in host behavior from time $t$ to time $t+1$ is defined as the sum of the host baseline behavior step and the microbiome-induced step. We denote by ${b}_{1}$ the first coordinate of the host behavior (corresponding to the *X*-axis along which the host moves) and define the host behavior at time $t+1$ as follows:

9 $$\,{b}_{1}\left(t+1\right)={b}_{1}\left(t\right)+\left({\sigma }_{A}\left(t\right)+M\left(t\right)\right)$$

### A second model for microbiome impact on host behavior

In this model the microbiome impact on host behavior is direct, and not through the reward circuitry of the host. The intuition for this model is that during the addiction and withdrawal, each microbial strain pulls the host towards its own coordinates (representing the optimal host behavior for that strain) in the microbiome-behavior space, and the host behavior is affected by the summation of host baseline trend and the microbiome impact projected on the examined behavioral domain ([0,1] along the *X*-axis).

First, we derive the direction of each microbial strain relative to the host behavior, by subtracting the host behavior coordinates from the strain’s feature-coordinates, and normalizing the resulting vector. Then all of the resulting vectors are averaged to obtain the overall microbiome impact direction. This average is weighted by the proportion of each microbial strain (${x}_{i}$), while each component is also multiplied by the strain’s impact strength (${d}_{i}$). In order to comply with our primary model, and investigate changes in behavioral pattern along a straight line, we derive the projection of the microbiome impact vector, on the *X*-axis. The shift in the host behavior is now determined by the sum of the host base-line step and the microbiome impact. In this sense, the parallel to Eq. 8 in this model is:

10 $$M\left(T\right)=\left[\mathop{\sum }\limits_{i=1}^{N}\left(\frac{{\vec{m}}_{i}-\vec{b}\left(T\right)}{{||}{\vec{m}}_{i}-\vec{b}\left(T\right){||}}\cdot {d}_{i}\cdot {x}_{i}\left(T\right)\right)\right]\cdot \left(\begin{array}{c}1\\ 0\\ \ldots \\ 0\end{array}\right)\cdot {\sigma }_{A}\left(T\right)$$

Similarly to the results of our primary model, here host-microbiome interactions lead to aggravated withdrawal as the microbiome impact strength increases and as the microbial diversity decreases (Supplementary Fig. 11).

### Reporting summary

Further information on research design is available in the Nature Portfolio Reporting Summary linked to this article.

## Supplementary information

Supplementary Information

Reporting Summary

## Data Availability

The simulation results that are presented in the figures are available at Zenodo (10.5281/zenodo.8041056).

## Code availability

The simulation code is available at Zenodo (10.5281/zenodo.8028697)^89^.
