# Supplementary material for: Evolutionary modeling suggests that addictions may be driven by competition-induced microbiome dysbiosis
Source: Commun Biol. 2023 Jul 26;6:782. doi: 10.1038/s42003-023-05099-0 (PMC10372008; doi:10.1038/s42003-023-05099-0)
Supplement: Supplementary file 1 — Supplementary Information [file 42003_2023_5099_MOESM1_ESM.pdf]

Evolutionary modeling suggests that addictions may be driven by competition-induced microbiome dysbiosis

Authors

Ohad Lewin-Epstein, Yanabah Jaques, Marcus W Feldman, Daniela Kaufer, Lilach Hadany*

Supplementary Figures

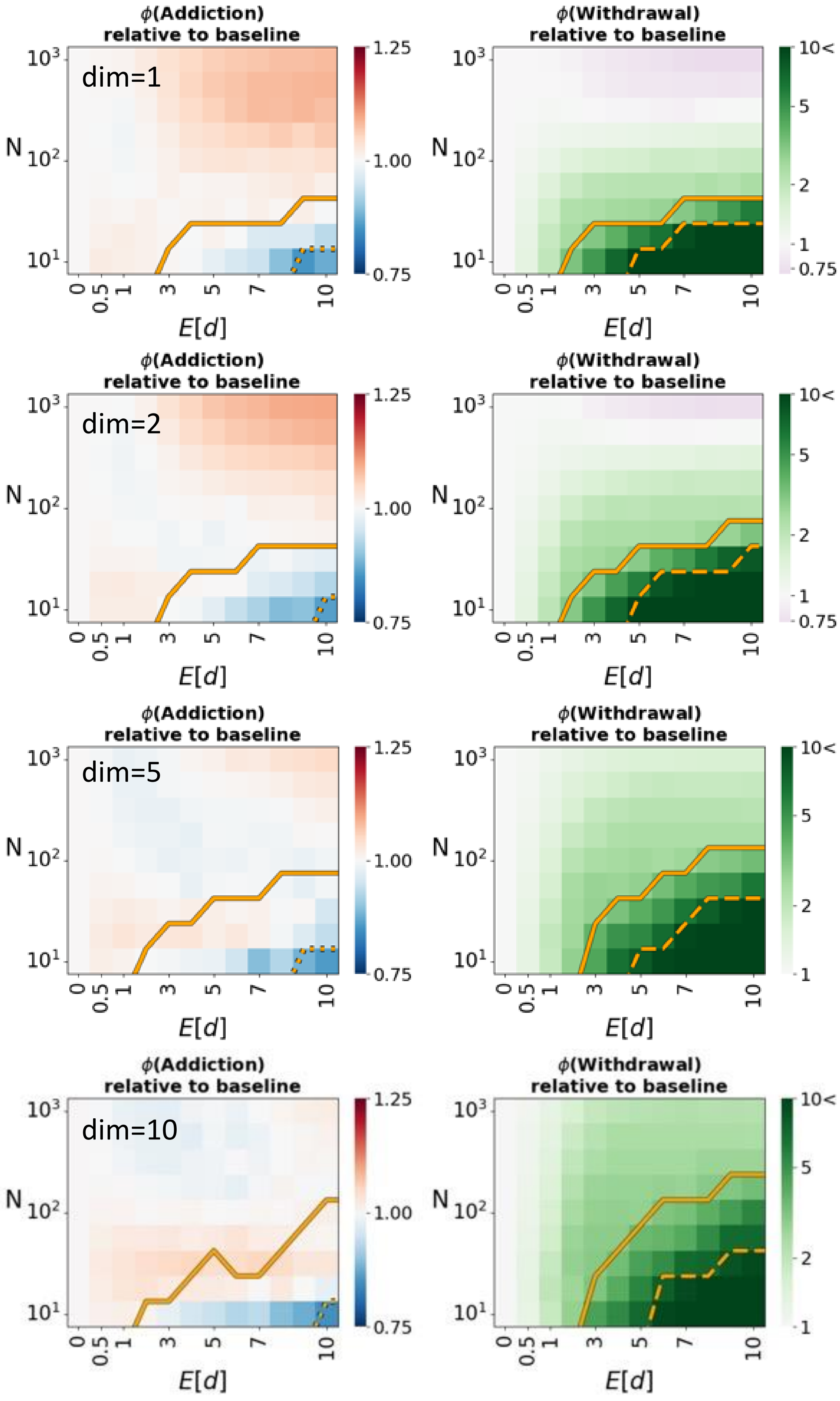
ϕ(Addiction)
relative to baseline
ϕ(Withdrawal)
relative to baseline
dim=1
dim=2
dim=5
dim=10
N
10^3
10^2
10^1
0
0.5
1
3
5
7
10
E[d]
1.25
1.00
0.75
10<
5
2
1
0.75

Supplementary Figure 1. Robustness of the model to the number of dimensions (D) in the microbiome-host behavior space. The color of each pixel in the heatmaps represent the fold increase or decrease in φ(Addiction) (left panels) and φ(Withdrawal) (right panels) relative to the baseline case of no microbiome effect, as functions of N and $E[d]$ for $D = 1$ (first row of panels), $D = 2$ (second row of panels), $D = 5$ (third row of panels) and $D = 10$ (last row of panels). Each pixel in the heatmaps presents the average of 500 simulations. The second row, where D=2, is the same as Fig. 3c,d in the main text.

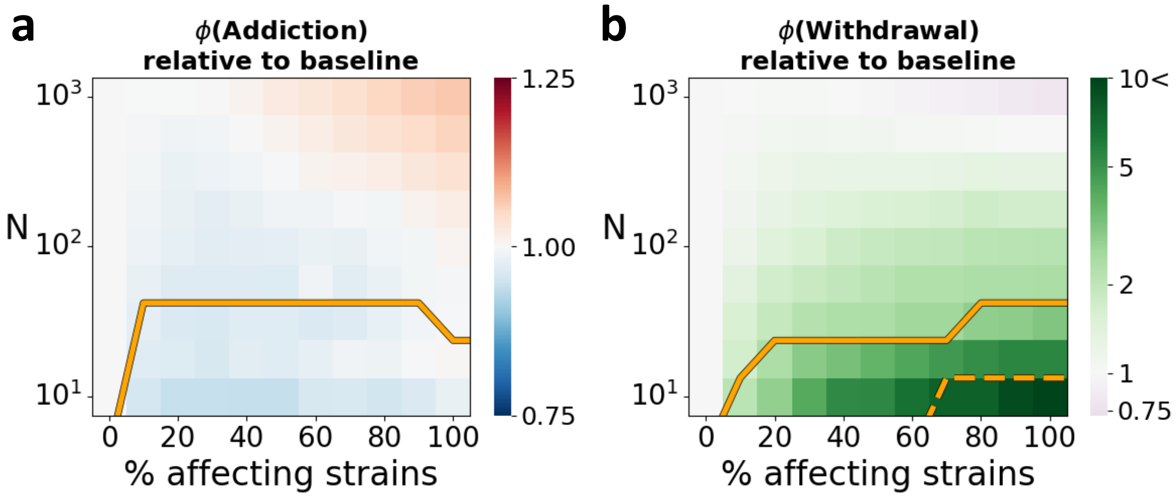

Supplementary Figure 2. The microbiome can strongly affect the addictive behavior, even when the majority of the strains do not affect the host behavior. The color pf each pixel in the heatmaps represent the fold increase or decrease in ϕ(Addiction) (a) and ϕ(Withdrawal) (b) relative to the baseline case of no microbiome effect, as functions of N and of the percentage of strains that can affect the host behavior. Each pixel in the heatmaps presents the average of 1,000 simulations. The figure is similar to Fig. 3e,f in the main text, except for the mean effect magnitude of the affecting microbes $(E[d])$ which, in the simulations presented in this figure, was set to 5, throughout. Below the solid lines in (a) more than 1% of the simulations do not reach the maximal addiction severity (R). Below the solid line in (b) the behavior in more than 1% of the simulations does not return to the initial state by the end of the simulation, and below the dashed line, it does not return in more than 20% of the simulations. $R = 0.7$.

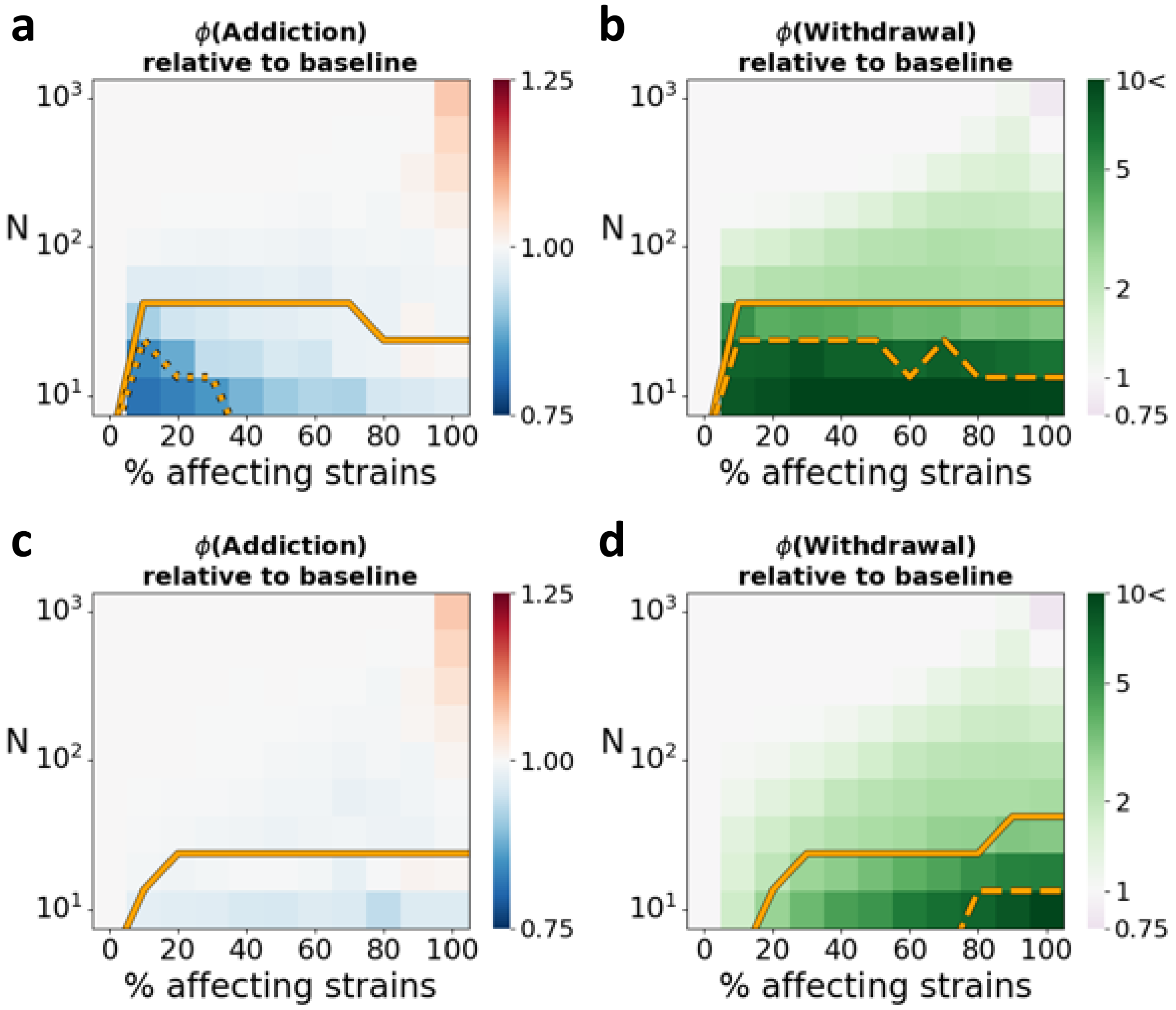

Supplementary Figure 3. The microbiome can strongly affect the addictive behavior, even when only part of the microbiome affects the host behavior, and including when the microbial production of the effect is costly. The color of each pixel in the heatmaps represent the fold increase or decrease in ϕ(Addiction) (a,c) and ϕ(Withdrawal) (b,d) relative to the baseline case of no microbiome effect, as functions of N and of the percentage of strains that can affect the host behavior. Each pixel in the heatmaps presents the average of 1,000 simulations. The figure is similar to Fig. 3e,f in the main text, but the simulations presented in this figure included a cost of 0.03 conferred by the microbes that had the ability to affect host behavior (see Methods, and equation 1). For the simulations that generated panels (a) and (b) we set $E[d] = \frac{5}{proportion\ of\ affecting\ strains}$ while for panels (c) and (d) we set $E[d] = 5$. Below the solid lines in (a,c) more than 1% of the simulations do not reach the maximal addiction severity (R), and below the dashed line more than 20% do not reach it. Below the solid line in (b,d) the behavior in more than 1% of the simulations does not return to the initial state by the end of the simulation, and below the dashed line, it does not return in more than 20% of the simulations. $R = 0.7$.

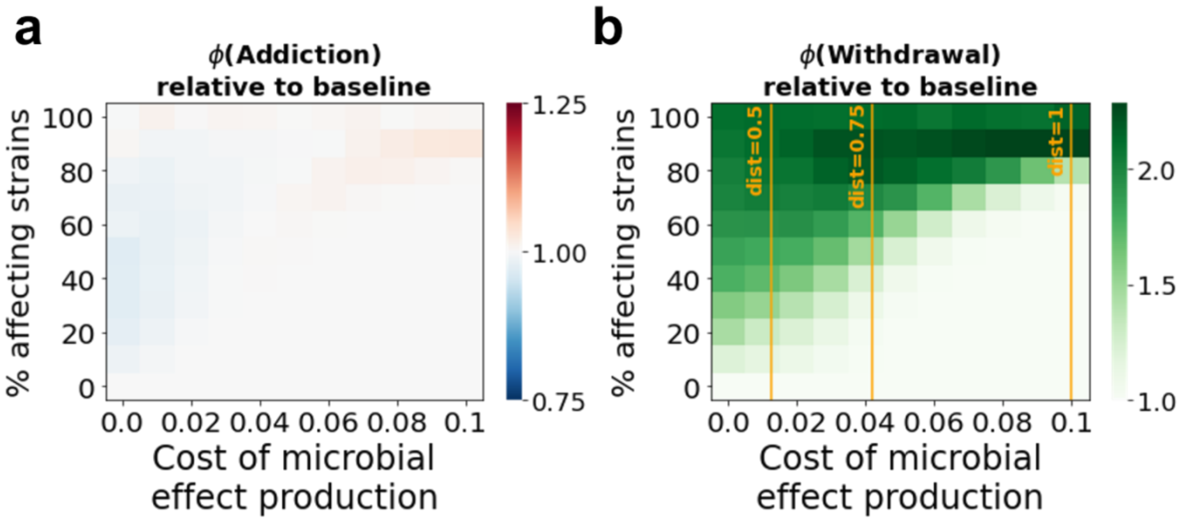

Supplementary Figure 4. Microbiome effect on host withdrawal can be significant even when only some of the strains can produce the effect, and even when this production is costly. The color of each pixel in the heatmaps represent the fold increase or decrease in ϕ(Addiction) (a) and ϕ(Withdrawal) (b) relative to the baseline case of no microbiome effect. Each pixel in the heatmaps presents the average of 1,000 simulations. The vertical lines in (b) indicate the distance advantage (in the microbiome-behavior-space) that an affecting microbe must have under optimal host behavior, relative to non-affecting strains, in order to compensate for the cost of producing a microbial effect. $R = 0.7, N = 100, E[d] = 5$.

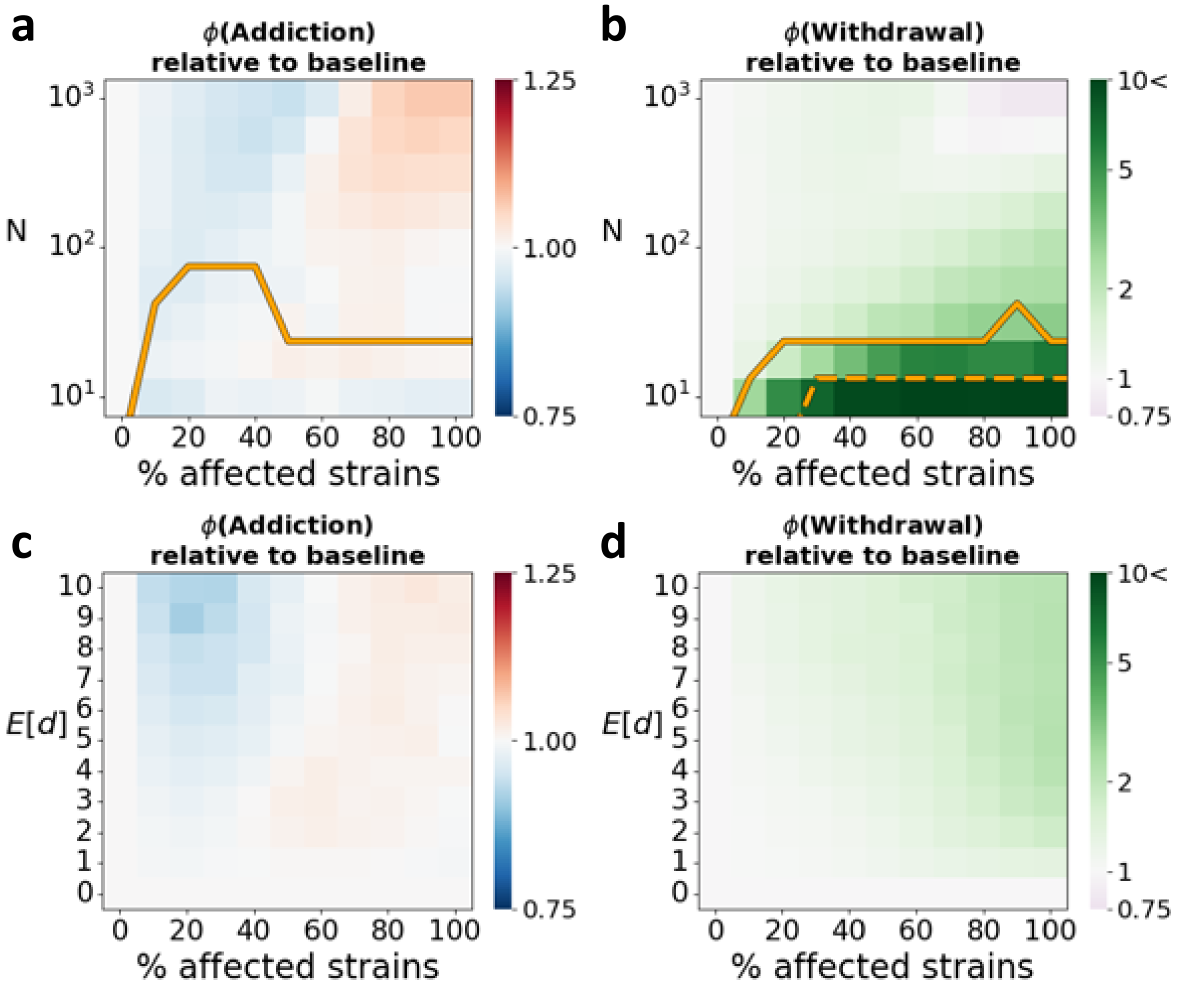

Supplementary Figure 5. Microbiome effect on host addiction and withdrawal may be significant even when only part of the microbiome is affected by the changes in the host behavior. The color of each pixel in the heatmaps represent the fold increase or decrease in ϕ(Addiction) (a,c) and ϕ(Withdrawal) (b,d) relative to the baseline case of no microbiome effect, as functions of the proportion of strains that are affected by the host changes in the behavior (x-axis), N (on the y-axis of panels a,b) and $E[d]$ (on the y-axis of panels c,d). Each pixel in the heatmaps presents the average of 1,000 simulations. In these simulations, the host factors that affect the strains' growth (r_i; equation 1 in Methods) were set to remain constant throughout the simulation. These strains (following the proportions indicated on the x-axis) were randomly chosen at the beginning of each simulation and their r_i value for the entire simulation was determined according to the distance between each strain's feature-coordinates, and the initial behavior coordinate (set at the origin). Below the solid line in (a) more than 1% of the simulations do not reach the maximal addiction severity (R). Below the solid line in (b) the behavior in more than 1% of the simulations does not return to the initial state by the end of the simulation, and below the dashed line, it does not return in more than 20% of the simulations. $R = 0.7$. In panels (a) and (b) $E[d] = 5$ while in panels (c) and (d) $N = 100$.

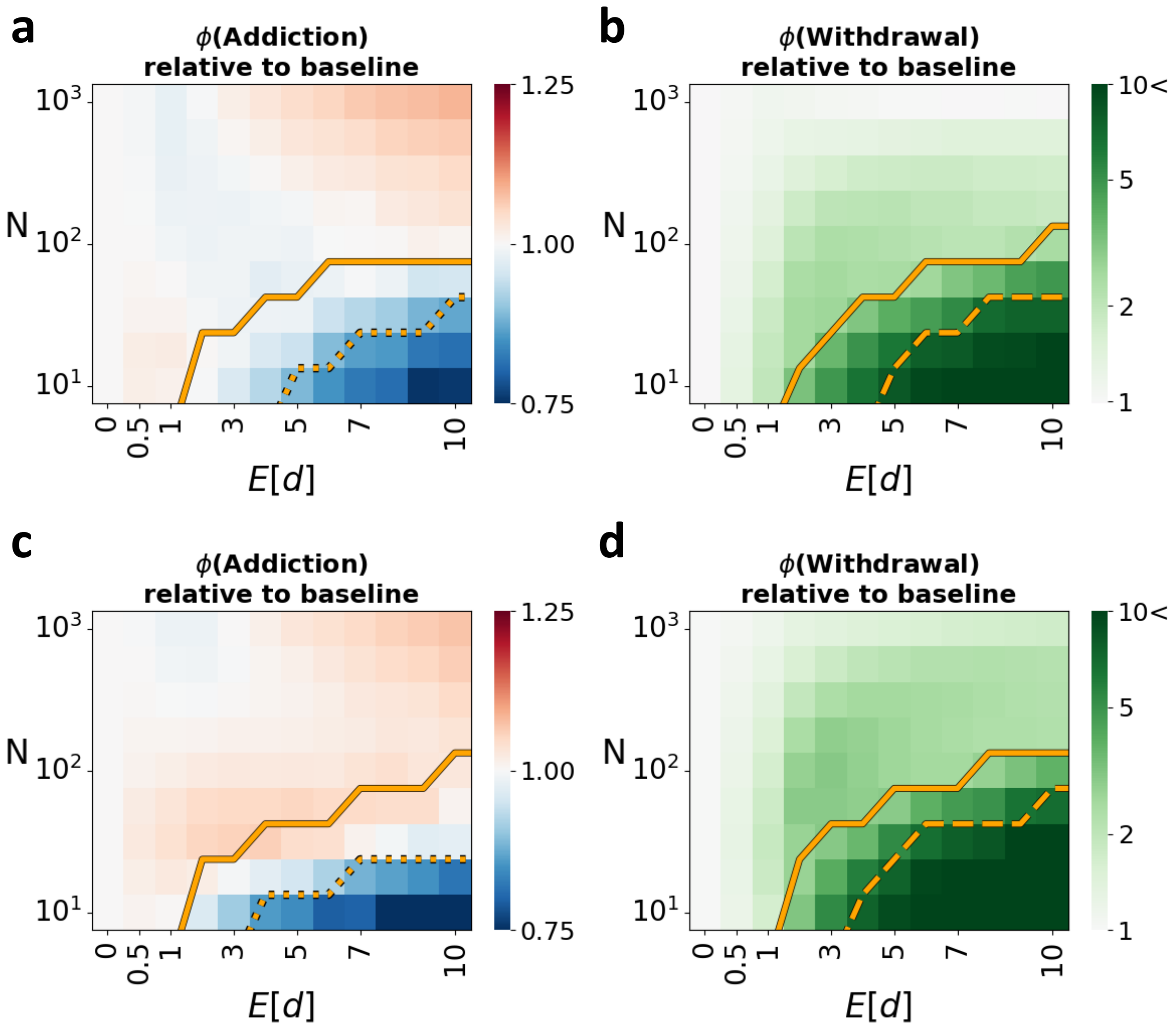

Supplementary Figure 6. Robustness of the results for a second modeling approach, based on generalized Lotka-Volterra equations. Using the generalized Lotka-Volterra model, described in section 2 of the Methods, the heatmaps present the fold increase or decrease in ϕ(Addiction) and ϕ(Withdrawal) relative to the baseline case of no microbiome effect, as functions of N and $E[d]$. The results are qualitatively similar to those in Fig. 3c,d in the main text. In the top row the inter-strain competition factor is $\delta = 0.5$, while for the bottom panels it is $\delta = 0.25$. Each pixel in the heatmaps presents the average of 1,000 simulations.

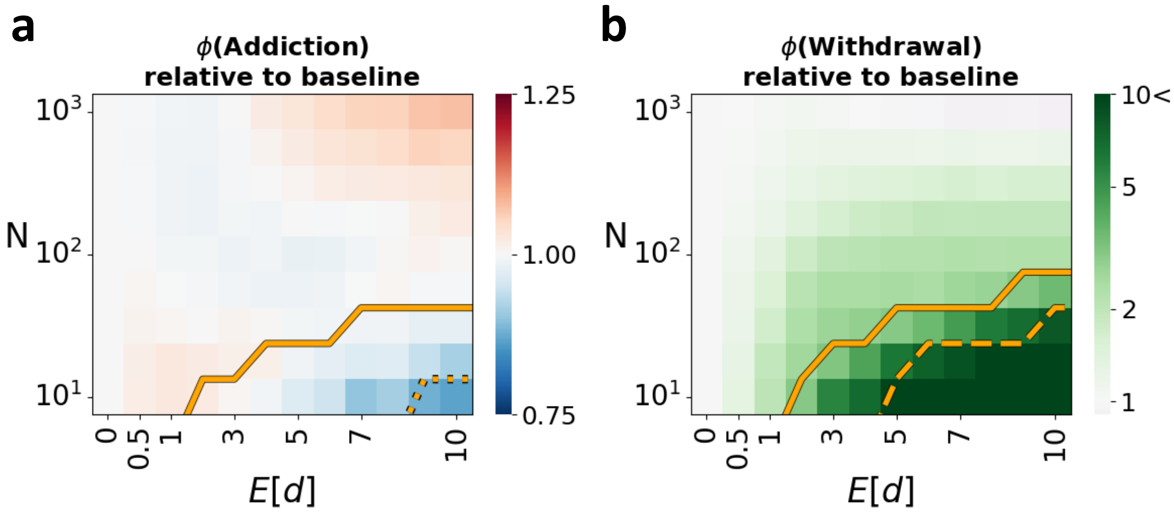

Supplementary Figure 7. Robustness of the results to analysis that includes a constant rate of strain-inflow. This figure is similar to Fig. 3c,d in the main text except that for this figure we kept the inflow of each strain at 10^{-9}, throughout the simulation. Thus for $N = 10$ the total inflow is $10 \cdot 10^{-9} = 10^{-8}$ and for $N = 1{,}000$ the total inflow is $1{,}000 \cdot 10^{-9} = 10^{-6}$. This is in contrast to Fig. 3c,d, where the total inflow was constant (10^{-8}) throughout the analysis. The results are very similar to Fig. 3c,d. Each pixel in the heatmaps presents the average of 500 simulations.

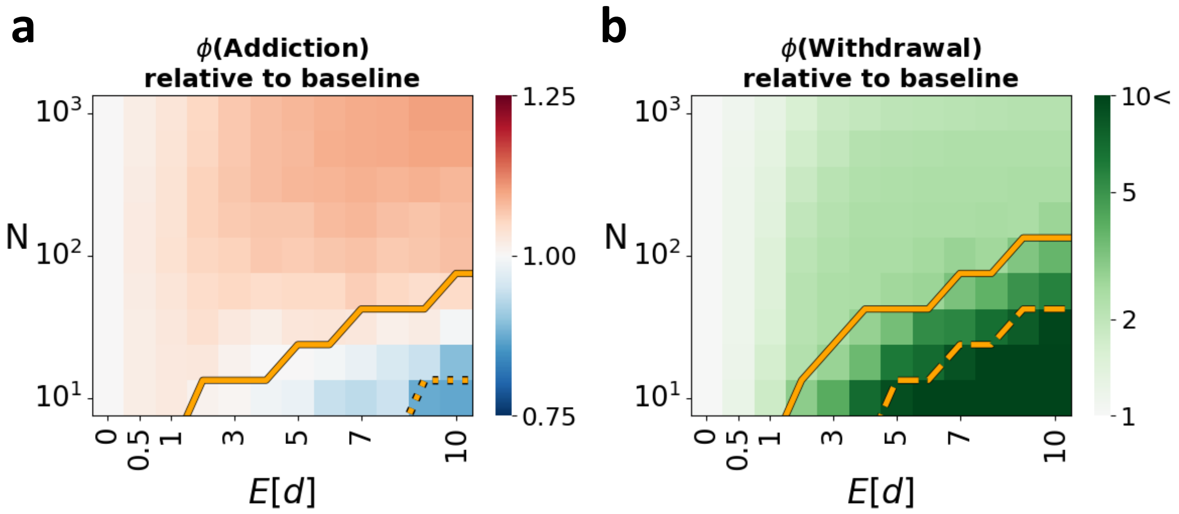

Supplementary Figure 8. Robustness of the results to absence of inflow. This figure is similar to Fig. 3c,d in the main text except that here we set the inflow rate to zero. Moreover, we enabled extinctions, by setting to zero the proportions of each strain that goes below 10^{-14}. Each pixel in the heatmaps presents the average of 500 simulations.

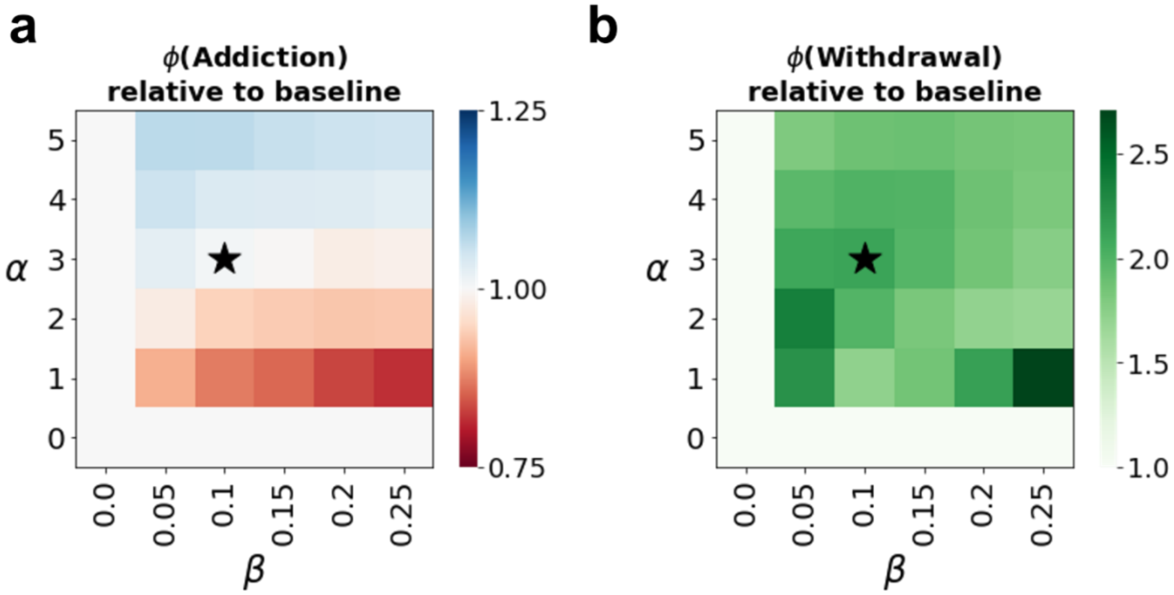

Supplementary Figure 9. Robustness of the results to the choice of parameters controlling the host-behavior impact on the microbes' growth. The color of each pixel in the heatmaps represent the fold increase or decrease in ϕ(Addiction) (a) and ϕ(Withdrawal) (b) relative to the baseline case of no microbiome effect, as functions of α and β (see Methods). Each pixel in the heatmaps presents the average of 1,000 simulations. Marked by star are the parameters that were used throughout the manuscript. $R = 0.7, N = 100, E[d] = 5$. It can be seen that while in the addiction stage, varying α and β yields bidirectional outcomes – shortening or prolonging the addiction, varying α and β does not change the trend of the withdrawal – the microbiome impact on host behavior leads to prolonged withdrawal.

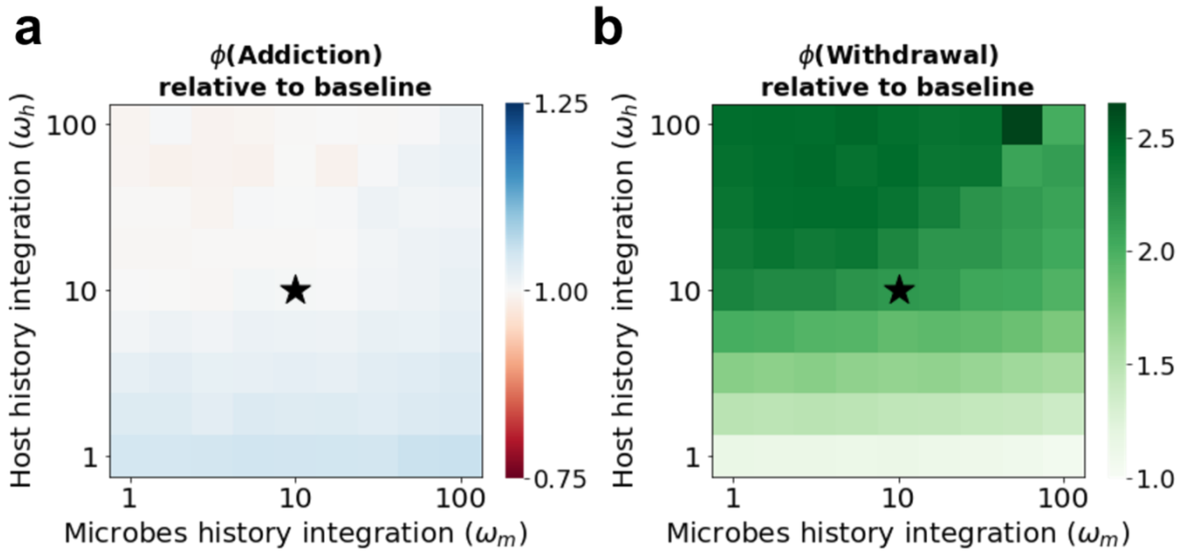

Supplementary Figure 10. Robustness of the results to the choice of the length of the time frame considered in the host and microbiome trajectories. The color of each pixel in the heatmaps represent the fold increase or decrease in ϕ(Addiction) (a) and ϕ(Withdrawal) (b) relative to the baseline case of no microbiome effect, as function of ω_h and ω_m (see Methods). Each pixel in the heatmaps presents the average of 1,000 simulations. Marked by star are the parameters that were used throughout the manuscript. $R = 0.7, N = 100, E[d] = 5$. There is almost no impact on the addiction stage (for the examined parameters), and some impact on the withdrawal, but all have the same trend - the microbiome impact on host behavior leads to prolonged withdrawal.

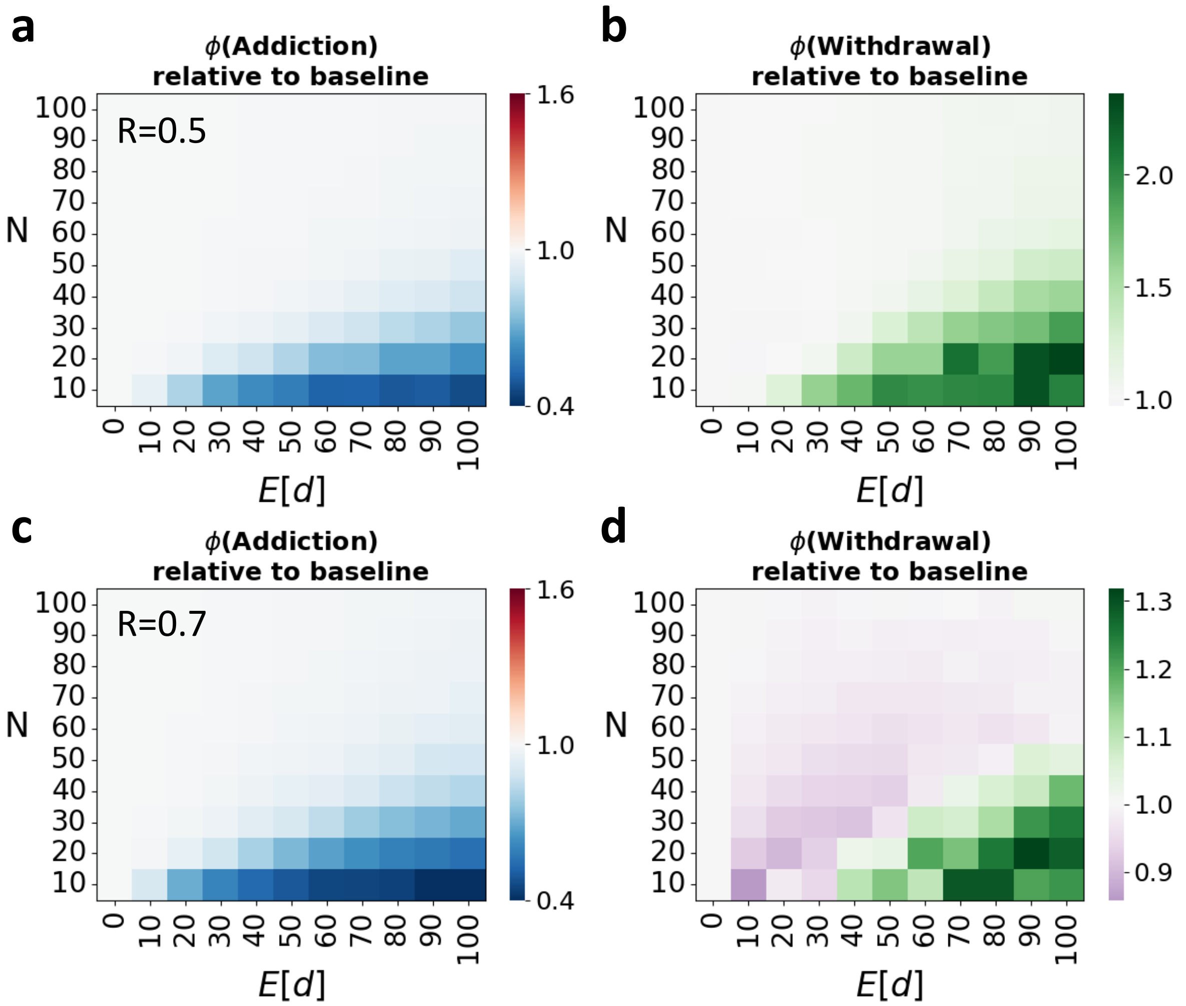

Supplementary Figure 11. Host-microbiome interactions and impact on host behavior under an alternative host-effect model, where each strain affects the host directly towards a behavior that is most beneficial for that strain (see Methods). The color of each pixel in the heatmaps represent the fold increase or decrease in ϕ(Addiction) and ϕ(Withdrawal) relative to the baseline case of no microbiome effect, as functions of N and $E[d]$, for addiction severity $R = 0.5$ (top row) and $R = 0.7$ (bottom row; similarly to the main text). Each pixel in the heatmaps presents the average of 1,000 simulations. Similarly to Figure 3c,d in the main text we find that the withdrawal is prolonged as the microbiome becomes less diverse and less rich (low N) and as the microbial impact strength increases ($E[d]$). For the addiction stage we find only a decrease in the ϕ(Addiction) generated by the host-microbiome interactions, unlike our primary model (Fig. 3c,d), for which the direction of the impact on the addictions (shortening or prolonging) depends on N and $E[d]$. Although this model and our primary model are different in the modeling approach of the microbiome-effect, note the difference in the $\mathrm{E}[d]$ scale between this figure and Fig. 3c,d in the main text.
